# Innovative Methylcellulose-Polyvinyl Pyrrolidone-Based Solid Polymer Electrolytes Impregnated with Potassium Salt: Ion Conduction and Thermal Properties

**DOI:** 10.3390/polym14153055

**Published:** 2022-07-28

**Authors:** Abdullahi Abbas Adam, Mohammed Khalil Mohammed Ali, John Ojur Dennis, Hassan Soleimani, Muhammad Fadhlullah Bin Abd. Shukur, Khalid Hassan Ibnaouf, Osamah A. Aldaghri, Moez A. Ibrahem, Naglaa F. M. Abdel All, Abubakar Bashir Abdulkadir

**Affiliations:** 1 Department of Fundamental and Applied Sciences, Universiti Teknologi PETRONAS, Seri Iskandar 32610, Malaysia; jdennis100@gmail.com (J.O.D.); hassan.soleimani@utp.edu.my (H.S.); mfadhlullah.ashukur@utp.edu.my (M.F.B.A.S.); abubakarbashir150@gmail.com (A.B.A.); 2 Centre of Innovative Nanoscience and Nanotechnology (COINN), Universiti Teknologi PETRONAS, Seri Iskandar 32610, Malaysia; 3 Department of Physics, Al-Qalam University Katsina, Katsina 820252, Nigeria; 4 Department of Physics, College of Science, Imam Mohammad Ibn Saud Islamic University (IMSIU), Riyadh 13318, Saudi Arabia; khiahmed@imamu.edu.sa (K.H.I.); odaghri@imamu.edu.sa (O.A.A.); 9@imamm.org (M.A.I.); nfabdelall@imamu.edu.sa (N.F.M.A.A.)

**Keywords:** methylcellulose, polyvinyl pyrrolidone, potassium carbonate, ethylene carbonate, solid polymer electrolytes

## Abstract

In this research, innovative green and sustainable solid polymer electrolytes (SPEs) based on plasticized methylcellulose/polyvinyl pyrrolidone/potassium carbonate (MC/PVP/K_2_CO_3_) were examined. The MC/PVP/K_2_CO_3_ SPE system with five distinct ethylene carbonate (EC) concentrations as a plasticizer was successfully designed. Frequency-dependent conductivity plots were used to investigate the conduction mechanism of the SPEs. Electrochemical potential window stability and the cation transfer number of the SPEs were studied via linear sweep voltammetry (LSV) and transference number measurement (TNM), respectively. Additionally, the structural behavior of the SPEs was analyzed using Fourier transform infrared spectroscopy (FTIR), field emission scanning electron microscopy (FESEM), X-ray diffractometry (XRD), and differential scanning calorimetry (DSC) techniques. The SPE film complexed with 15 wt.% EC measured a maximum conductivity of 3.88 × 10^−4^ Scm^−1^. According to the results of the transference number examination, cations that record a transference number of 0.949 are the primary charge carriers. An EDLC was fabricated based on the highest conducting sample that recorded a specific capacitance of 54.936 Fg^−1^ at 5 mVs^−1^.

## 1. Introduction

Recently, solid-state electrolytes (SSEs) have arisen as a technology of significant research and commercial relevance for the storage of electrical energy. Solid polymer electrolytes (SPEs) are a class of SSEs that comprise an inorganic salt dispersed in a polymer host material [1]. Currently, SPEs are getting particular attention due to a variety of factors, including the development of high-performance materials, better safety concerns, and innovative applications [2,3,4,5]. By modifying their chemistry, several SPEs now achieve ionic conductivity equivalent to those of liquid electrolytes at room temperature. Additional advantages of SPEs over the liquid type include mechanical stability, excellent film formation with superior ionic conductivity, and self-standing ability [6,7,8,9,10,11,12,13,14].

To meet the continued demand for low-cost, high-performing, and high-safety electrolyte materials, there is an increasing demand to come up with novel materials for PEs or modify the current ones. Among these materials, special attention is given to SPEs due to their fascinating properties. In comparison to liquid and gel electrolytes, SPEs have several advantageous characteristics, including a lack of leakage; excellent thermal, electrochemical, and volumetric stabilities; absence of solvents; and low volatility. Other properties that are attracting increased interest in SPEs include high potential stability, superior mechanical strength, longer cycle life, simple processing and fabrication, lightweight, high automation process, high energy density, flexibility, and the ability to be configured in a number of different geometries [15].

Cellulose (the most abundant natural biopolymer) and its derivatives are widely used in SPEs, primarily for their biodegradability, low cost, and environmental friendliness [4,16]. Among the cellulose derivatives, methylcellulose (MC) is a distinctively water-soluble semicrystalline polymer with an ionic conductivity of around 10^−11^ Scm^−1^ [17,18]. However, this magnitude is insufficient for practical use in SPEs’ technology. By modifying the chemistry of MC, it has shown promising ionic conductivity in the amorphous phase, allowing for better ion transport inside the polymer [6,19]. According to previous reports [20,21], blending polymers improves the electrochemical and physicochemical properties of the electrolyte system by expediting the ion migration process and providing additional complexation sites within the polymer matrix. Specifically, blending MC with another polymer has been an effective way to augment the amorphous nature of MC-based SPEs [22,23,24].

In addition to natural-based polymers such as MC, there has also been an extensive investigation on SPEs based on synthetic polymers such as polyvinyl alcohol (PVA) [25,26,27], polyvinylidene fluoride (PVDF) [28,29], polyethylene oxide (PEO) [30,31], and polyvinyl pyrrolidone (PVP) [32,33,34,35]. Among these polymers, PVP has recently received much interest due to its amorphous structure, biocompatibility, high glass transition temperature, high solubility in water, and the potential to form complexes with various salts [32,33,34,35,36]. The PVP pyrrolidine groups are responsible for good ionic mobility while the carbonyl groups are responsible for the formation of various complexes with different salts [37]. Therefore, mixing PVP with other synthetic or natural polymers will expectedly result in a plasticized polymer blending with high amorphousness and increased ion mobility in the amorphous phase.

Due to their rapid cation mobility and strong ionic conductivity, potassium salts have shown their suitability for use in SPEs, among alkali metal salts. Because potassium is a big cation, it is seldom entrapped by polymeric networks; hence, K^+^ movement within the polymer matrix requires less activation energy [38]. In this study, K_2_CO_3_ was chosen as a conducting salt due to its strong propensity to dissociate because of its large ion size and high ionic conductivity-free ions. In addition to its high ion content, superior thermal stability, non-volatility, non-combustibilaty, low viscosity, and environmental friendliness, it also has a low viscosity [39,40]. Furthermore, K_2_CO_3_ was selected owing to its high plasticizing action, hydrophilic characteristics, and inexpensive cost. The K^+^ from K_2_CO_3_ is a cation with a tiny radius; thus, tiny cations may be readily dissociated and transferred as charge carriers or ions in SPEs [41].

The solution casting procedure is a well-known technique for preparing stand-alone films and is less complicated compared to other methods such as electrochemical deposition [42], sol-gel [43], and citrate-gel combustion [44]. To obtain SPE with great mechanical, thermal, and electrical qualities, it is critical to choose polymers that are compatible with one another and with the salt [45]. Thus, in this work, we report the synthesis of a new SPE based on an MC and PVP blend doped with potassium carbonate (K_2_CO_3_) using the conventional solution casting technique. The concentration of K_2_CO_3_ was varied to investigate the effect of K^+^ in the solid electrolyte films. To further improve the conductivity of the prepared samples, the highest conducting sample was plasticized with EC at various concentrations, and the effects of EC on conductivity, potential stability, and crystallinity were studied.

## 2. Materials and Methods

### 2.1. Materials

MC (viscosity of 4000 cP) and PVP (M_w_ of 40,000 gmol^−1^) were procured from Sigma Aldrich, Malaysia, through Evergreen Chemicals Supply (Selangor Malaysia). K_2_CO_3_ (M_w_ of 138.21 gmol^−1^) and EC (M_w_ of 88.06 gmol^−1^) were purchased from R and M Chemicals. All chemicals were used as supplied without any treatment. DI water was used as the only solvent throughout the experiment.

#### 2.1.1. Synthesis of MC/PVP Polymer Blend

**Figure 1:**
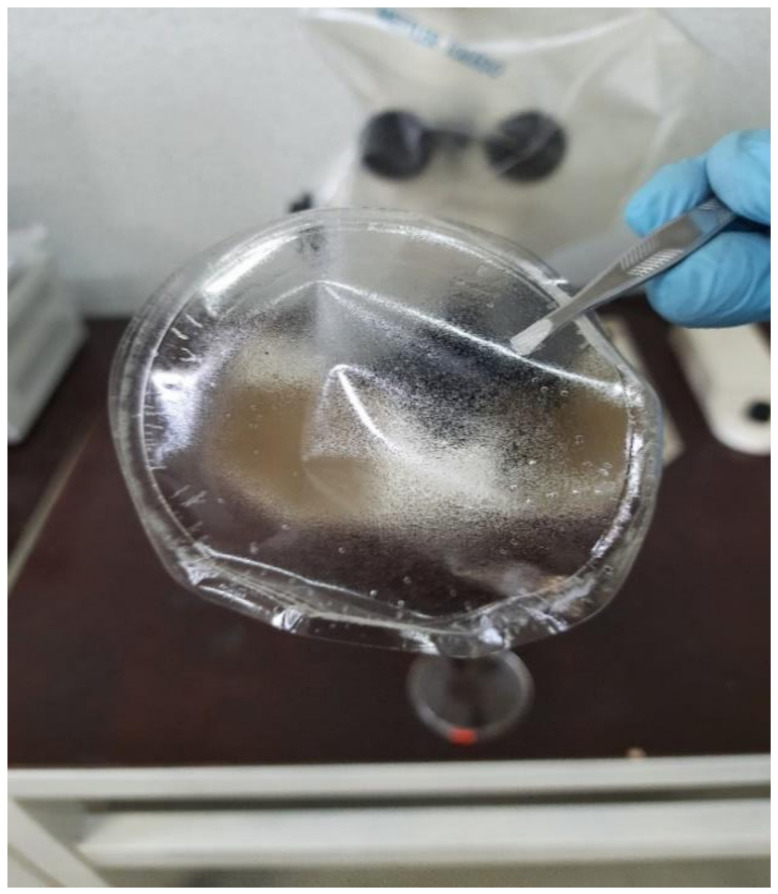
MC/PVP SPE film.

A required mass of MC (1.0–0.5 g) was dissolved in DI water (100 mL) and stirred at 50 °C for a few hours until fully dissolved. The PVP (0–0.5 g) was added to the MC solution and stirred at room temperature for a few hours to obtain a homogenous solution. To ensure total and homogenous blending, the solution was then sonicated for 1 h followed by 30 min of additional stirring at room temperature. Using this approach, six different samples containing different ratios of MC to PVP (100:0, 90:10, 80:20, 70:30, 60:40, and 50:50) were prepared. Samples containing higher concentrations of PVP were not prepared because stand-alone films could not be synthesized due to their extremely poor mechanical strength. Each solution was cast on a petri dish and covered with a filter paper (to avoid contamination) to dry at room temperature for 3 to 5 days. Prepared films were stored in a desiccator. To easily identify the prepared samples, they were labeled MP0, MP10, MP20, MP30, MP40, and MP50 for the various concentrations of PVP in the samples. A typical image of a prepared MC/PVP SPE film is shown in Figure 1. It is worth stating that a stand-alone film was not obtained when the concentration of PVP was higher than that of MC. Rather, the film stuck firmly to the petri dish, as can be seen in Figure S1. Furthermore, the prepared SPEs were extremely flexible and durable against different forms of manipulations, as can be seen in Figure S2.

#### 2.1.2. Synthesis of MC/PVP/K_2_CO_3_ SPE

**Figure 2:**
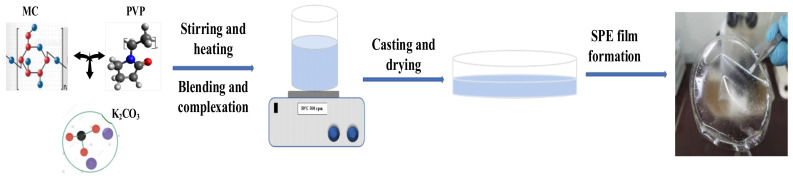
Flow diagram showing the preparation of MC/PVP/K_2_CO_3_ SPEs.

To the most amorphous MC/PVP electrolyte (MP50), K_2_CO_3_ was added to prepare the MC/PVP/K_2_CO_3_ SPE films in a similar way to that in which MC/PVP was prepared. In brief, MC (0.5 g) was dissolved in 100 mL of DI water under stirring at 50 ℃. When it was fully dissolved, an equal mass of PVP was added and the mixed solution was stirred at room temperature until a homogenous solution was obtained. Next, 5–25 wt.% of K_2_CO_3_ was added to five different MC/PVP solutions and each solution was stirred for 1 h. The solutions were then sonicated for 1 h followed by stirring on a hot plate for 30 min (at room temperature) to ensure the complete dispersion of K_2_CO_3_. Each solution was then poured onto a petri dish, covered with filter paper, and stored at room temperature to dry gradually. To ensure nearly equal film thickness, the volumes of all electrolyte samples cast on petri dishes were maintained at 40 mL. Dried films were peeled off and stored in a desiccator for further drying before characterization. Prepared samples containing different concentrations of potassium salt were labeled as MPK5, MPK10, MPK15, MPK20, and MPK25. The flow diagram depicting the synthesis of the prepared SPE is presented in Figure 2.

#### 2.1.3. Synthesis of MC/PVP/K_2_CO_3_/EC SPE

To prepare plasticized samples, EC was incorporated into MPK20, which was the SPE with the highest ionic conductivity. The salted samples were prepared according to the explanation in Section 2.1.2 above. When the polymer-salt complex solution is fully dissolved, 5–25 wt.% (interval of 5) EC plasticizer was added to the solution and stirred for 20 min before sonication for another 20 min. Finally, the solution was further stirred for 30 min (at room temp) and allowed to settle for 1 h to expel air bubbles before casting. Each solution was cast on petri dish and dried at room temperature. Dried films were peeled off and stored in a desiccator for further drying before characterization. To denote the samples containing different amounts of EC, the following codes were used: MPKE5, MPKE10, MPKE15, MPKE20, and MPKE25.

#### 2.1.4. EDLC Fabrication

The supercapacitor (EDLC) was fabricated by sandwiching the most conductive electrolyte (MPKE15) between two activated carbon (AC) electrodes produced and packed inside a CR2032 coin cell. To begin the preparation of the electrode, 1 g of polyvinylidene fluoride (PVdF) was dissolved in 30 mL of N-methyl pyrrolidone (NMP) and stirred at 50 °C. After 20 min, the solution was cooled to room temperature while being constantly stirred. Meanwhile, 0.5 g of carbon black (acetylene black) was added and mixed for 2 h at room temperature. Finally, 6.5 g of AC was added gradually, and the mixture was stirred until it was completely dissolved. The slurry was ultrasonically treated for 30 min (to guarantee homogeneity) followed by further stirring on a hot plate at room temperature for a further 1 h and then cooled to room temperature. The produced slurry was coated on nickel foams that were previously pressed and weighed. The coated nickel foams were dried at 50 °C for 24 h and then re-weighed to calculate the mass of the deposited slurry. AC electrodes of equivalent slurry mass were matched for the production of the EDLC.

### 2.2. Characterization of Electrolyte Samples

Fourier transform infrared (FTIR) studies of the prepared films were conducted on an FTIR spectrometer (Bruker Instruments, model Aquinox 55, Germany) in the 4000–400 cm^−1^ range using KBr pellets with a scanning resolution of 4 cm^−1^. To analyze the crystal structure for prepared films, X-ray diffraction (XRD) was carried out using a 40 kV Bruker D8 Advance X-ray diffractometer with a current of 40 mA using a Ni-filtered Cu Kα graphite monochromator (Ś = 1.5406 Å). The study of surface morphology of the synthesized samples was examined using field emission scanning electron microscopy (FESEM) [46,47]. Differential scanning calorimetry (DSC) (model DSC Q2000 V24.11, Oberkochen, Germany) was used to investigate the thermal behavior of the SPEs. The glass transition temperature (*T_g_*) of the samples was measured at a heating rate of 10 °Cm^−1^ from −50 °C to 190 °C in a nitrogen (N_2_) environment.

### 2.3. Electrochemical Studies of Solid Electrolytes

The electrochemical studies were performed to analyze the ionic conductivity and operating potential window of each polymer electrolyte. The measurement was performed in the two electrodes’ system using AUTOLAB/AUT51018. The SPE sample was sandwiched between two blocks of a stainless-steel sample holder with a 3.142 cm^2^ surface area that were positioned opposite each other. Transference number measurement (TNM) was performed using a digital DC power supply (V&A Instrument DP3003).

### 2.4. EDLC Characterization

#### Cyclic Voltammetry (CV)

The electrochemical characteristics of EDLC were investigated using eight different scan rates: 5 mVs^−1^, 10 mVs^−1^, 20 mVs^−1^, 40 mVs^−1^, 80 mVs^−1^, 100 mVs^−1^, 200 mVs^−1^, and 500 mVs^−1^ in the potential range between −0.9 V and 0.9 V . To characterize the EDLC, a potentiostat (AUTOLAB/AUT51018) equipped with Nova 2.1.4 software was employed. Using the following formula [3], the specific capacitance (*Csp*) of EDLC cells was determined.

(1) $$C_{sp}=\frac{1}{2mv\left(V_{f}-V_{i}\right)}\;\int_{V_{i}}^{V_{f}}I\;\left(V\right)dV$$

where *I(V)dV* represents the CV’s area as calculated by the OriginPro 2021’s program integration function. The parameters *m* and *v* are the average mass of active materials and scan rate, respectively. In this study, the initial and final applied potentials (*V_i_* and *V_f_*) were −0.9 V and 0.9 V, respectively.

## 3. Results and Discussion

### 3.1. Structural Analysis

#### 3.1.1. FTIR Studies

**Figure 3:**
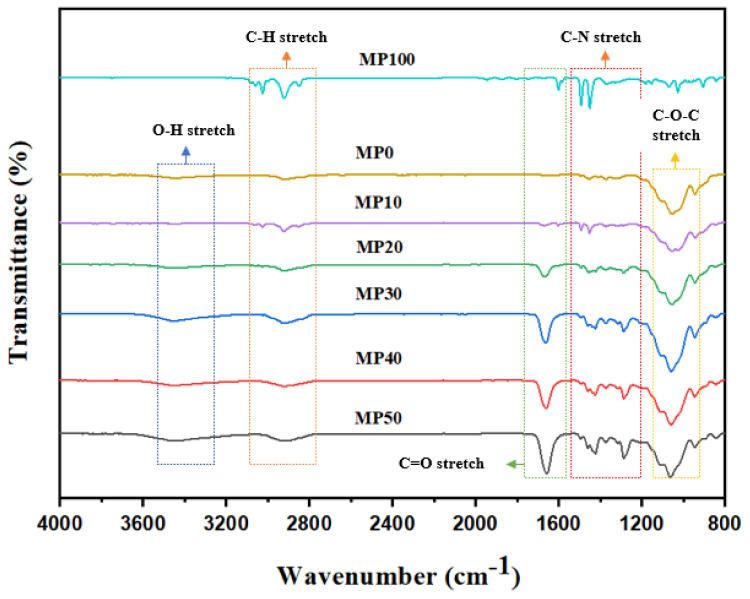
FTIR of MC/PVP polymer blend.

“FTIR is a direct method to distinguish molecular interactions by monitoring the band shifts of certain functional groups” [48]. The FTIR spectra of the MC/PVP polymer blend within the range of 800–4000 cm^−1^ are presented in Figure 3 and the band assignments are shown in Table 1. For pure MC, absorption peaks were observed at 3479 cm^−1^, 2907 cm^−1,^ and 1057 cm^−1,^ which correspond to O–H stretching, C–H (in CH_2_) stretching, and C–O–C stretching, respectively [7,21,46]. Similarly, pure PVP exhibited distinctive peaks at 2918 cm^−1^, 1614 cm^−1,^ and around 1500 cm^−1,^ which were attributed to C–H stretching, C=O stretching, and C–N stretching, respectively [32,47,48]. As reported by Hamsan et al. [16], the interaction between two polymers in a blend system is indicated by the shifting of the FTIR peaks of the functional groups containing oxygen atoms (such as hydroxyl and ether groups). Thus, we investigated the blending of MC with PVP by observing the band shift in the polymer blend systems.

When MC blends with another polymer, the shift in the FTIR peaks occur at the oxygen atom-containing functional groups of MC [21]. In the MC/PVP polymer blend, the hydrogen atoms in PVP form hydrogen bonds with the oxygen atoms of the hydroxyl groups of MC (or vice versa). Here, we may assume that the oxygen atoms in MC’s O–H groups formed short-ranged hydrogen bonds with the corresponding hydrogen atoms of PVP. For MP10, the O–H stretch shifted to 3436 cm^−1^ due to the hydrogen bond interaction between the two polymers. As the concentration of PVP increased, the O–H band of the polymer blend shifted further to a higher wavelength region (as shown in Table S1). These results clearly indicated that MC and PVP blended homogenously, and the amorphous structure of the polymer blend increased with increasing PVP concentration, as confirmed by XRD patterns.

It was evidently clear that a complex system was formed when salt and the polymer matrix interacted. The presence of complete complexation between salt cations and polymer functional groups is indicated by a decrease in transmittance intensity and a change in band position [6]. As a result of the electrostatic synergy between the functional group and the salt cation, the vibration inside the polar group was diminished. Changes in peak location imply principally the change in the state of the electron dispersion or hybridization inside the chemical bond. Typically, a reduction in peak intensity indicates a decrease in the number of functional groups linked with the molecular bond (per unit volume) [49,50].

**Figure 4:**
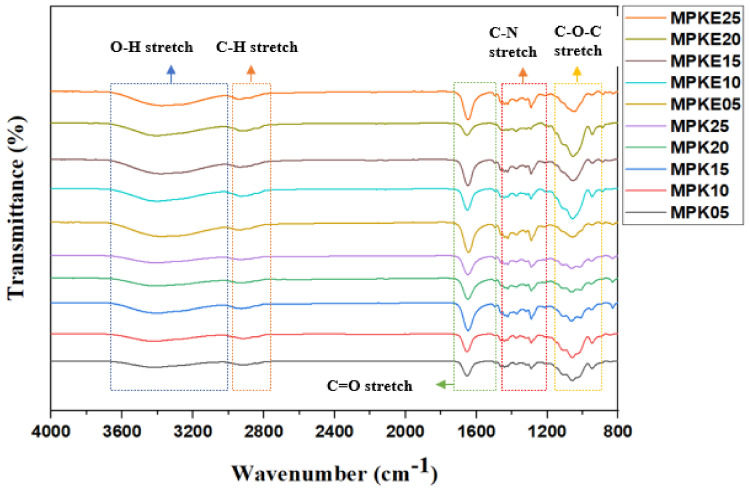
FTIR of plasticized and non-plasticized MC/PVP/K_2_CO_3_ SPEs.

**Figure 5:**
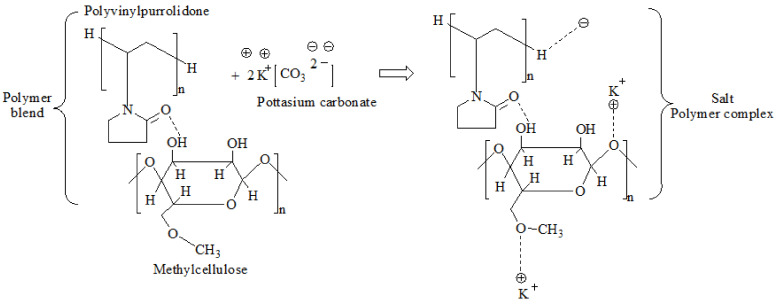
Schematic representation of MC/PVP/K_2_CO_3_ polymer-salt complexation.

Here, when MP50 was doped with varying amounts of K_2_CO_3_ (Figure 4), the OH stretch not only broadened, it equally downshifted, indicating the development of a polymer-salt complex [21,51,52]. The shift in the OH band spectrum was attributed to the reciprocating between the K^+^ hopping mechanism and MC/PVP host segmental motion. The potassium atom in K_2_CO_3_ readily dissociated from the parent molecule to create K^+^ and wandered within the polymer matrix. As a result of its free movement within the polymer structure, the K^+^ facilitated conduction inside the polymer matrix. The anticipated polymer-salt complexation for the prepared SPE is shown in Figure 5. According to Hamsan et al. [47], an increase in salt content increases the number of cations that may interact with the oxygen atom of the hydroxyl or carbonyl groups of MC/PVP, leading to an increase in ionic conductivity. However, the IR spectrum corresponding to the OH stretch nearly disappeared for MPK25. This denoted the formation of neutral ion pairs via ion recombination. The production of neutral ion pairs may decrease the quantity of charge carriers in a polymer matrix, hence lowering its ionic conductivity [47].

On a closer look, the FTIR spectra of the EC plasticized samples demonstrated that the spectrum of each MPKE film was almost identical to that of MPK50. However, the intensities of certain peaks (OH and COC) varied appreciably. C=O in the EC may have interacted with the alkyl group of MC/PVP molecular chains, which might have contributed to the band shift observed at the OH and COC regions. A potential reason is that EC may have interacted with K_2_CO_3_, hence altering K_2_CO_3_’s interaction with the MC/PVP skeleton. Generally, ion dissociation is facilitated when a plasticizer is added to the polymer-salt complex. This enables additional ions to interact with the polymer’s functional groups [48]. The increase in EC concentration causes a change in the transmittance intensity due to ion-dipole complexation resulting from an increased number of K^+^. Increasing EC content increases K_2_CO_3_ dissolution. The increased coordination between K^+^ and the C=O segment of EC results in enhanced dissociation of K^+^, thereby generating a weak bond that promotes electron conduction via the delocalized pi system [53,54]. In addition, these weak bonds provide alternative routes for K^+^ to enter the coordinating sites of the MC/PVP backbone.

Plasticizers are believed to play a role as spacers between polymer molecules by creating linkages with them as a result of dipole couplings between polar groups in the plasticizer [48,55]. As a result of the presence of EC, the Columbic force between K_2_CO_3_ cations and anions was lowered. Consequently, more salts were dissociated into free mobile ions, increasing the ion density and the conductivity [56,57]. However, the addition of more than 15 wt.% EC reduces the conductivity of the SPE. This is due to the displacement of K_2_CO_3_ inside the polymer-salt complexes by EC molecules, which cause the salt to recrystallize, resulting in a decrease in conductivity.

#### 3.1.2. XRD Analysis

**Figure 6:**
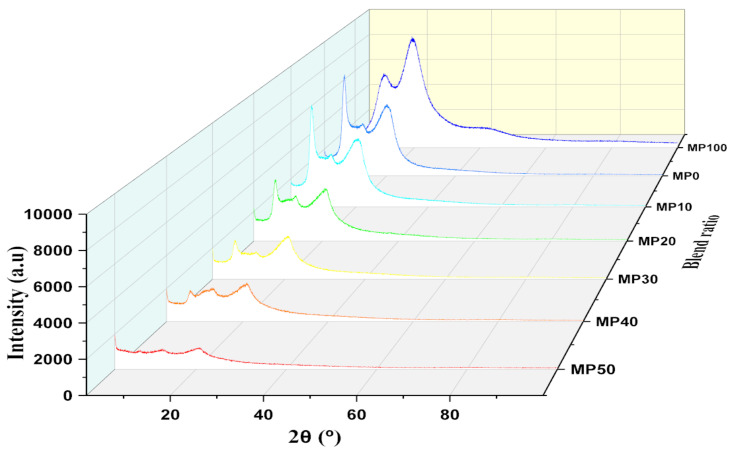
XRD pattern of the MC/PVP polymer blend.

An XRD study was employed to investigate the crystallinity of MC/PVP SPE films and to analyze the formation of the MC/PVP blend system [58]. The structure and phase patterns of MC/PVP SPEs are presented in Figure 6. According to the literature [59,60], MC has a prominent broad peak around 19–22°, which is a characteristic of its semicrystalline structure resulting from hydrogen bonding at both the intermolecular and intramolecular levels. In most cases, a relatively sharp peak can also be observed in the MC film at 8°. This peak is attributed to a sequence of trimethyl cellulose present in MC [59]. Because PVP (MP100) is completely amorphous, crystalline peaks are barely observed. However, two broad amorphous bands are usually noticed around 2θ = 11° and 22° [61]. From the XRD patterns obtained in this study, MC peaks were observed at 2θ = 7.8° and 20.2° while amorphous PVP exhibited two hollows at 2θ = 10.9° and 19.6°. This result is consistent with some of the reported literature [7,61,62]. The XRD pattern showed a steady decline in the intensity of the MC peak upon the addition of varying concentrations of PVP. Similarly, the hollows observed in PVP equally disappeared as the MC/PVP composite was being formed. The increase in the amorphous phase of the system confirmed the successful blending of MC with PVP. With a further increase in the amount of PVP in the MC precursor, the intensity became more obvious until an optimum result was obtained for the MP50 sample. Thus, MP50 was taken as the optimum sample for further studies.

### 3.2. Morphological Analysis

#### FESEM Analysis

**Figure 7:**
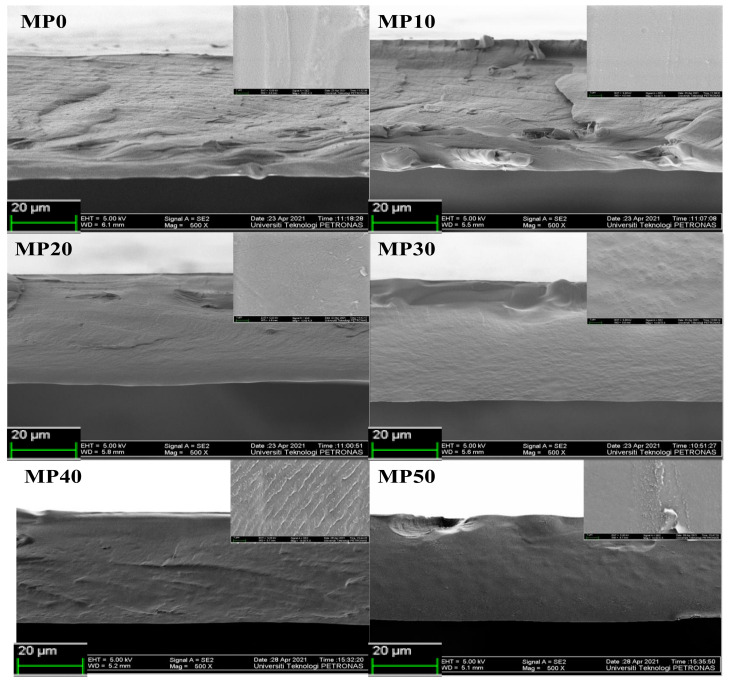
FESEM micrographs of MC/PVP polymer blend.

FESEM micrographs can provide an understanding of the compatibility of the various components of a composite SPE and their interfaces. In addition, the surface examination enables one to understand the alterations in the structural and electrical characteristics of SPEs [52]. According to Hamsan et al. [24], a cross-sectional view of an FESEM micrograph is an excellent way to study the miscibility of polymer blend systems. Figure 7 shows cross-sectional FESEM images of MC/PVP polymer blend systems with the top view shown as an inset. As the micrographs demonstrate, the pure MC (MP0) film exhibited a homogenous, smooth surface, which is a characteristic property of semicrystalline MC. For various MC/PVP blend systems (MP10–MP50), smoother and denser cross-sectional images were obtained due to the incorporation of PVP. The polymer blend system’s smoothness was seen to increase as the concentration of PVP increased, mainly due to the amorphous nature of PVP. All FESEM images showed no evidence of phase separation, which showed that MC and PVP were homogenously blended. Moreover, smoother FESEM images indicate better blending between the polymers and improved amorphous phase. Since the improved amorphous section provided better ion transfer pathways, MP50 seemed to show better morphology compared to other films. This observation was consistent with the XRD and FTIR results previously explained. Similar patterns of increasing amorphousness at higher contents of PVP were seen in the surface view of the FESEM images (shown in insets). Because pure PVP cannot be peeled off from a petri dish, the FESEM of the MP100 film analyzed on a petri dish substrate is shown in Figure S1b. The numerous holes seen on the surface of the film were due to trapped air as the PVP stuck to the substrate, thus forming bubbles.

### 3.3. DSC Analysis

**Figure 8:**
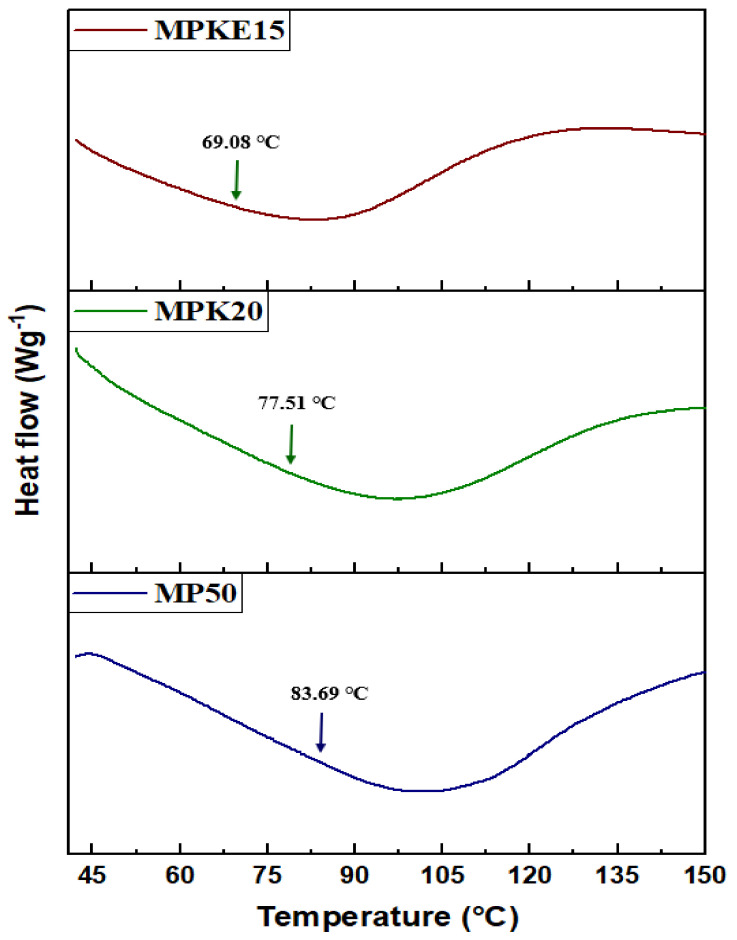
DSC thermograms of selected MC/PVP-based SPEs.

According to Genier et al. [63], the low ionic conductivity of SPEs is caused by the high glass transition temperature and high polymer crystallinity of the host polymers. The thermal behavior of MP50, MPK20, and MPKE15 was examined by DSC analysis. Figure 8 illustrates the alterations in Tg for the analyzed samples’ record between 41–150 °C. All DSC thermograms depicted an endothermic peak with a single step transition, which means that MC and PVP are miscible with each other [21]. The Tg of MP50 was recorded at 83.69 °C. Upon doping with 20 wt.% K_2_CO_3_, the Tg was lowered to 77.51 °C. The decrease in the Tg value suggested that the polymer salts’ segment became softer in the amorphous phase structure [64], hence improving the segmental mobility of the polymer-salt complex. Moreover, the reduction in Tg of the polymer host was similarly associated with the increase in ionic conductivity of SPEs. The introduction of the salt into the MC/PVP system weakened the dipole-dipole interactions between the MC/PVP chains. In turn, this allowed the ions to travel freely across the polymer chain network when an electric field was applied, hence enhancing the conductivity [65,66].

EC was selected for this study because of its high dielectric constant and low vapor pressure. With the addition of EC, it was envisaged that many charge carriers would localize along with the mobile ions, thereby improving the SPE’s ionic conductivity [67]. As expected, the DSC thermogram of MPK15 showed a significant decrease in the *T_g_* value (9.08 °C). This is because the inclusion of EC aided in boosting the amorphous fraction of SPEs as well as improving the polymer segmental mobility. Feng et al. [68] also reported a similar pattern where they observed that both LiClO_4_ and EC cause a shifting of an endothermic peak of the PEO-based SPE to a lower temperature.

### 3.4. Electrochemical Studies

#### 3.4.1. EIS Studies

**Figure 9:**
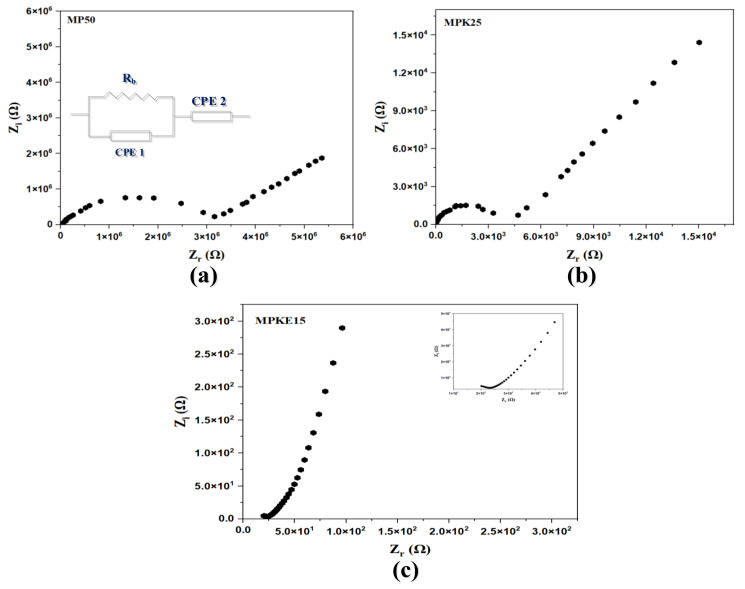
Nyquist plots of selected MC/PVP-based SPE with associated ECC. (**a**) MP50, (**b**) MPK25 and (**c**) MPKE15.

As stated earlier, AUTOLAB/AUT51018 (potentiostat/galvanostat) was used to study the ionic conductivity of the polymer blend electrolytes over a range of frequencies from 10^−2^ Hz to 10^5^ Hz and at room temperatures [48]. The Nyquist plots of selected samples (MP50, MPK20, and MPKE15) together with their corresponding electrical circuit model (EEC) are shown in Figure 9. The EEC approach was employed to examine the EIS because it is straightforward and provides a complete view of the system [69]. As seen in the inset, the impedance diagrams are represented by an equivalent circuit composed of a charge transfer resistance (*R_b_*) in a parallel configuration with the first constant phase element (CPE1) in the high-frequency area and in a series arrangement with the second constant phase element (CPE2) in the low-frequency region. According to Nofal et al. [38], the impedance due to the CPE ($Z_{CPE}$) can be expressed in terms of the CPE capacitance (C) and angular frequency (ω) using Equation (3).

(2) $$Z_{CPE}=\frac{1}{C\omega^{\rho }}\left[cos\left(\frac{\pi \rho }{2}\right)-isin\left(\frac{\pi \rho }{2}\right)\right]$$

In the above equation, ρ defines the EIS deviation from the imaginary axis. The real and imaginary impedance ($Z_{r}$ and $Z_{i}$) associated with the EEC are given as:

(3) $$Z_{r}=\frac{R_{b}^{2}C_{1}\omega^{\rho_{1}}cos\left(\pi \rho_{1}/2\right)+R_{b}}{2R_{b}C_{1}\omega^{\rho_{1}}cos\left(\pi \rho_{1}/2\right)+R_{b}^{2}C_{1}^{2}\omega^{2\rho_{1}}+1}+\frac{cos\left(\pi \rho_{2}/2\right)}{C_{2}\omega^{\rho_{2}}}$$

(4) $$Z_{i}=\frac{R_{b}^{2}C_{1}\omega^{\rho_{1}}sin\left(\pi \rho_{1}/2\right)}{2R_{b}C_{1}\omega^{\rho_{1}}cos\left(\pi \rho_{1}/2\right)+R_{b}^{2}C_{1}^{2}\omega^{2\rho_{1}}+1}+\frac{sin\left(\pi \rho_{2}/2\right)}{C_{2}\omega^{\rho_{2}}}$$

The parameters $C_{1}$ and $C_{2}$ represent the CPE1′s capacitance (related to bulk of electrolytes) and CPE2′s capacitance (related to electrode-electrolyte interface), respectively. Similarly, $\rho_{1}$ and $\rho_{2}$ represent the offset from the real and imaginary axes, respectively.

The Nyquist plots in Figure 9 show a typical representation of an EDLC, which shows a semicircle inclined at angle *θ* with the real axis at a high-frequency region and a spike at a low-frequency region. The high-frequency semicircle emanates from the electrode-electrolyte interface while the angle of inclination emanates from the ions’ relaxation time. Equation (6) was employed to compute the ionic conductivity of the synthesized SPEs from the bulk resistance [70,71]. The bulk resistance is found from the intercept of the Nyquist plot with the real axis.

(5) $$\sigma =\frac{t}{R_{b}A}$$

Here, *t* stands for the thickness of the film (in cm), *R_b_* (in Ω) is the film’s bulk resistance, and *A* is the contact area of the electrode-electrolyte interface (in cm^2^). Table 1a presents the ionic conductivity calculated for different combinations of MC and PVP. Based on the Nyquist plots of the prepared films (Figure S3 in the Supplementary Materials), the bulk resistance decreased as the concentration of PVP decreased. This was due to the plasticizing effect of PVP, which increased the amorphous structure of the polymer blend, as shown in the XRD and FESEM analyses. Additionally, Figures S4 and S5 show that the bulk resistances of the SPE decreased appreciably upon doping with various concentrations of K_2_CO_3_ and EC. The increase in ionic conductivity with increasing K_2_CO_3_ up to 20 wt.% (Table 1b) could be attributed to CO_3_^2-,^ which could have plasticized the –C=O⋯K⋯O=C– quasi-cross-linking structure in the prepared SPE. A further increase in K_2_CO_3_ above 20 wt.% caused a sudden decline in ionic conductivity, possibly due to salt ion agglomeration in the polymer matrix [47].

**Table 1:** (a) Ionic conductivity of MC/PVP polymer blend, (b) ionic conductivity of MC/PVP/K_2_CO_3_ SPE, and (c) ionic conductivity of MC/PVP/K_2_CO_3_/EC SPE.

S/N	Sample	Film Thickness × 10^−3^ (cm)	Bulk Resistance (Ohm)	Ionic Conductivity (Scm^−1^)
**a**				
1.	MP0	9.3	8.97 × 10^7^	3.30 × 10^−11^
2.	MP10	9.5	5.31 × 10^7^	5.71 × 10^−11^
3.	MP20	9.7	5.14 × 10^7^	6.01 × 10^−11^
4.	MP30	9.9	3.33 × 10^7^	9.45 × 10^−11^
5.	MP40	9.4	1.56 × 10^7^	1.92 × 10^−10^
6.	MP50	9.2	2.98 × 10^6^	9.83 × 10^−10^
**b**				
1.	MPK5	2.33	4.46 × 10^4^	1.66 × 10^−7^
2.	MPK10	1.77	2.84 × 10^4^	1.98 × 10^−7^
3.	MPK15	2.16	2.13 × 10^4^	3.23 × 10^−7^
4.	MPK20	2.21	4.83 × 10^3^	1.46 × 10^−6^
5.	MPK25	2.11	4.46 × 10^4^	1.51 × 10^−7^
**c**				
1.	MPKE5	2.62	3.13 × 10^2^	2.66 × 10^−5^
2.	MPKE10	2.91	4.94 × 10^1^	1.87 × 10^−4^
3.	MPKE15	2.89	2.37 × 10^1^	3.88 × 10^−4^
4.	MPKE20	2.74	6.90 × 10^1^	1.26 × 10^−4^
5.	MPKE25	2.69	2.16 × 10^2^	3.98 × 10^−5^

When a low-molecular-weight plasticizer is added to a polymer-salt complex, more salt dispersion occurs, which increases the number of charge carriers and boosts the mobility of the polymer-salt complex [22]. In this work, EC (5–25 wt.%) was added to enhance not only the ionic conductivity but also the potential window of the MPK20 system. As seen in Table 1c, the ionic conductivity improved as EC concentrations increased until an optimum conductivity of 3.88 × 10^−4^ Scm^−1^ was attained for the 15 wt.% added sample. The increased conductivity was a result of the EC creating new channels for ions to flow through. Additionally, EC may diminish the Coulombic interaction involving cations and anions. More salts were dissociated into free mobile ions, resulting in an increase in the quantity of ions in the SPE matrix. However, the addition of 20 wt.% or more EC lowered ionic conductivity owing to the displacement of the host polymer by EC molecules inside the salt complexes, resulting in recrystallization of the salt and consequent conductivity reduction.

#### 3.4.2. LSV Studies

**Figure 10:**
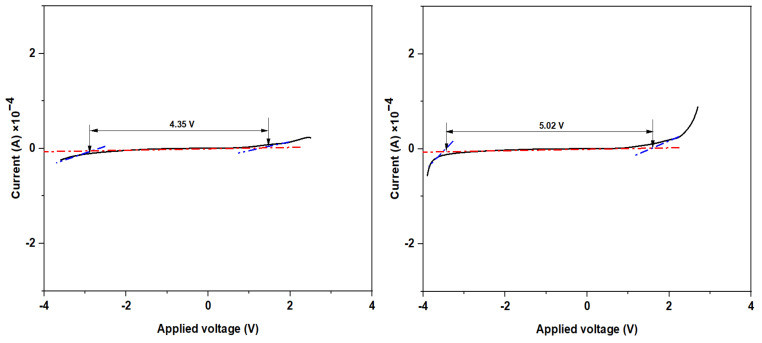
LSV curves of MPK20 and MPKE15 SPE.

The electrochemical stability window (ESW) is a critical parameter in SPE technology, particularly for device applications. The LSV approach is used to obtain an approximate breakdown voltage of an electrolyte [72]. Before conducting a charge-discharge cycle test on any device, the electrochemical stability of the sample must be determined because the breakdown voltage is essential for preventing electrolyte destruction. Figure 10 shows the operational stability window of MPK20 and MPKE15 samples investigated in an AUTOLAB/AUT51018 workstation at a potential range of −2–4 V at a scan rate of 10 mVS^−1^. According to Aziz et al. [52], an electrochemical potential window of 1.27 V is sufficient for a biopolymer electrolyte to be used in EDLC. In this work, the plasticizer-free and EC plasticized samples (MPK20 and MPKE15) recorded a high potential window of 4.35 V and 5.02 V, respectively. Considering the LSV curve of MPKE15, a considerable faradaic current did not appear in the potential range of −3.44 V to 1.57 V. This signifies that the electrolyte system was electrochemically stable across the specified range of potentials [73]. However, when the potential rose beyond 1.57 V, the current increased significantly due to electrolyte breakdown, particularly near the inert electrode’s surface. This was due to the decomposition of the SPE.

#### 3.4.3. TNM Measurement

**Figure 11:**
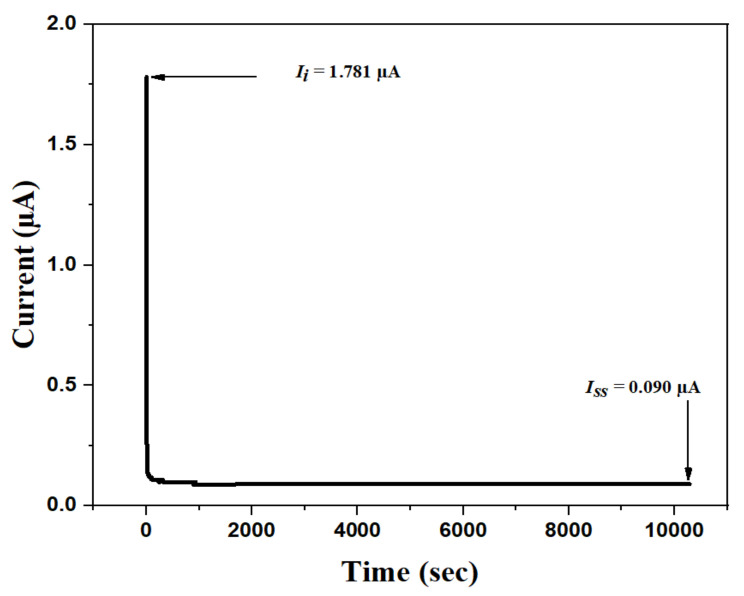
TNM plot of MPKE15 SPE.

In order to determine if an SPE is suitable for usage in an EDLC, the TNM test may be used. A DC potential was used in this approach, which established the relationship between current and time. At the onset, a high initial current (*I_i_*) of 1.781 A was observed, which was due to the contribution of both electrons and ions from an operating voltage of 0.2 V applied to the constructed cell setup. Researchers found that ions are the primary charge carriers in high-conducting electrolyte systems, whereas electrons are the secondary charge carriers [74]. This means the ions contribute more to conductivity. Figure 11 shows the current vs. time plot for the most conducting electrolyte (MPKE15) to discover which charge carrying species are the most prevalent.

Because of the equilibrium between ion diffusion and ion drift at the stainless-steel electrode, a dramatic decrease in current was shown in the polarization plot. As a result, electrons were the only species capable of passing through. Therefore, the steady-state current (*I_ss_* = 0.090 µA) owing to the electron was reached when all ions in the system were reduced to zero. In this study, the electron and ion transference numbers (*t_el_* and *t_ion_*) were determined by using Equations (6) and (7).

(6) $$t_{ion}=\frac{I_{i}-I_{ss}}{I_{i}}$$

(7) $$t_{el}=1-t_{ion}$$

According to Equation (5), the *t_le_* value is 0.051, whereas the *t_ion_* value is 0.949 (obtained from Equation (6). Obviously, the high *t_ion_* value obtained, which is near to the ideal value of one, demonstrates the ionic nature of the charge transfer mechanism inside the SPE. To better appreciate the results obtained in this study, we compared the performance of MPKE15 with some related work previously reported. As can be seen in Table 2, this work outperformed previous works in terms of collective electrochemical properties.

### 3.5. Device Study

#### CV Analysis

**Figure 12:**
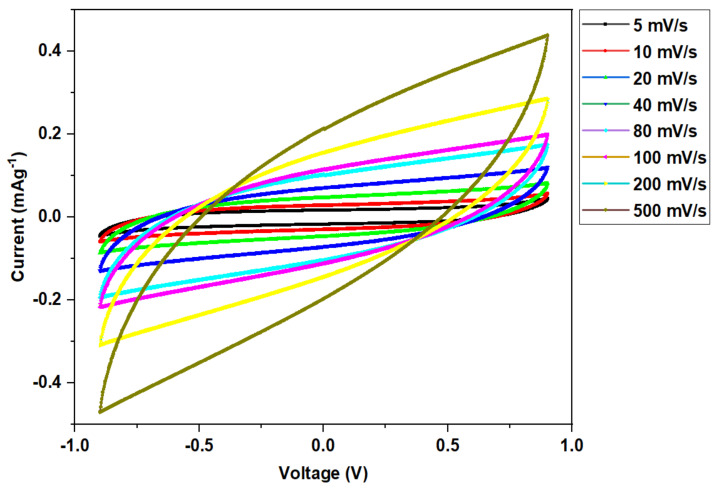
The CV profile for the fabricated EDLC cell at various scan rates.

Figure 12 illustrates the effect of a scan rate on the primary characteristic of the CV profile with no noticeable redox peak throughout the voltage range. The CV displays a leaf-like pattern at every scan rate. An ideal EDLC is defined by the presence of a rectangular CV electrochemical characteristic [78]. In essence, however, the primary characteristics of CV obtained in this study were altered owing to the nature of the electrode surface (porosity), which caused variations in internal resistance. This change in CV response influenced both the performance and efficiency of the EDLC [3]. Furthermore, the absence of redox peaks in the CV profile demonstrated the occurrence of a charge-storing capability through a non-faradaic process, which is a fundamental property of a true EDLC. In accordance with this method, both cations’ and anions’ adsorption and desorption occurred at the electrode surfaces; in other words, intercalation and deintercalation processes were absent [79].

The CV values at various scan rates are shown in Table 3, where the greatest value is observed at a low scan rate and declines progressively as the scan rate rises. In theory, at a low scan rate, a stable double-layer charge occurs at the interface due to the adsorption of ions [80]. Obviously, a nearly perfect plateau obtained at low scan rates is due to the formation of a broad diffusion layer at the interface as a result of ion adsorption. This phenomenon also results in low ohmic resistance at lower scan rates. At a fast scan rate, on the other hand, a narrow, diffusion layer is generated, resulting in a low capacitance [3].

## 4. Conclusions

In this work, MC/PVP/K_2_CO_3_-based SPE with added EC as plasticizer showed excellent electrochemical properties and, therefore, its viability as a green and sustainable replacement electrolyte. The SPE system was also able to match the performance of some existing green and sustainable SPEs. Various methods, such as XRD, FTIR, EIS, SV, and TNM, were used to examine the polymer samples’ structural and electrical characteristics. Molecular interactions between PVP and PVC were seen in the changing FTIR band peaks, which indicate a successful blending of the two polymers. Crystallinity was reduced when the PVP content was increased in the MC/PVP blend system. The presence and shift of identified functional groups in the FTIR study demonstrated the creation of an MC/PVP/K_2_CO_3_ complex and that EC interacted with this complex. The MPK20 doped with 15% EC (MPKE15) had the greatest room temperature conductivity of 3.88 × 10^−4^ Scm^−1^ with a potential window of 5.02 V. According to the LSV study, the breakdown of the film occurred at potentials greater than 5.02 V, and the sample exhibited stability throughout a broad range of potential windows. As per the TNM study, the *t_ion_* value for MPKE15 was 0.949 but the *t_el_* value was 0.051. Using the optimum sample (MPKE15), an EDLC was fabricated and the CV profile of the EDLC recorded a specific capacitance of 54.936 Fg^−1^ at 5 mVs^−1^.

## Figures and Tables

**Table 2:** Comparison of electrochemical performance of selected SPEs.

Polymer-Salt Complex	RT Ionic Conductivity (Scm^−1^)	Potential Window (V)	Ion Transference Number	Ref.
CS/DX/NH_4_PF_6_/glycerol	3.0 × 10^−4^	1.5	0.96	[75]
CS/MC/NH_4_NO_3_/glycerol	1.31 × 10^−4^	1.87	0.93	[22]
CS/MC/NH_4_I	1.93 × 10^−4^	2.10	0.93	[76]
MC/PC/K_3_PO_4_/glycerol	3.0 × 10^−4^	4.19	-	[6]
MC/PC/NH_4_Cl/ZnO	3.13 × 10^−4^	4.55	-	[66]
DX/CS/NafT/glycerol	6.10 × 10^−5^	2.55	0.99	[69]
PVA/CS/NH_4_SCN	1.36 × 10^−5^	2.25	0.72	[77]
MC/PVP/K_2_CO_3_/EC	3.88 × 10^−4^	5.02	0.949	This work

RT = room temperature.

**Table 3:** Variation of specific capacitance at different scan rates for the fabricated EDLC.

Scan Rate (mVs^−1^)	Specific Capacitance (Fg^−1^)
5	54.936
10	45.849
20	36.065
40	26.797
80	18.936
100	16.600
200	10.897
500	5.923

## Data Availability

The data presented in this study are available on request from the corresponding author. The data are not publicly available due to privacy.

## Supplementary Materials

The following supporting information can be downloaded at https://www.mdpi.com/article/10.3390/polym14153055/s1. Figure S1: (a) Pure PVP film stuck to the petri dish, (b) FESEM image of pure PVP film on petri dish substrate. Figure S2: Images of SPEs under different manipulations. Figure S3: Nyquist plots of MC/PVP polymer blend samples. Figure S4: Nyquist plots of other MC/PVP/K_2_CO_3_ SPEs. Figure S5: Nyquist plots of other MC/PVP/K_2_CO_3_/EC SPEs. Table S1: Band assignment for MC/PVP polymer blend. References [81,82,83,84,85] are cited in the Supplementary Materials.
