# Supplementary material for: Innovative Methylcellulose-Polyvinyl Pyrrolidone-Based Solid Polymer Electrolytes Impregnated with Potassium Salt: Ion Conduction and Thermal Properties
Source: Polymers (Basel). 2022 Jul 28;14(15):3055. doi: 10.3390/polym14153055 (PMC9370478; doi:10.3390/polym14153055)
Supplement: Supplementary file 1 [file polymers-14-03055-s001.zip › polymers-1811608-supplementary.pdf]

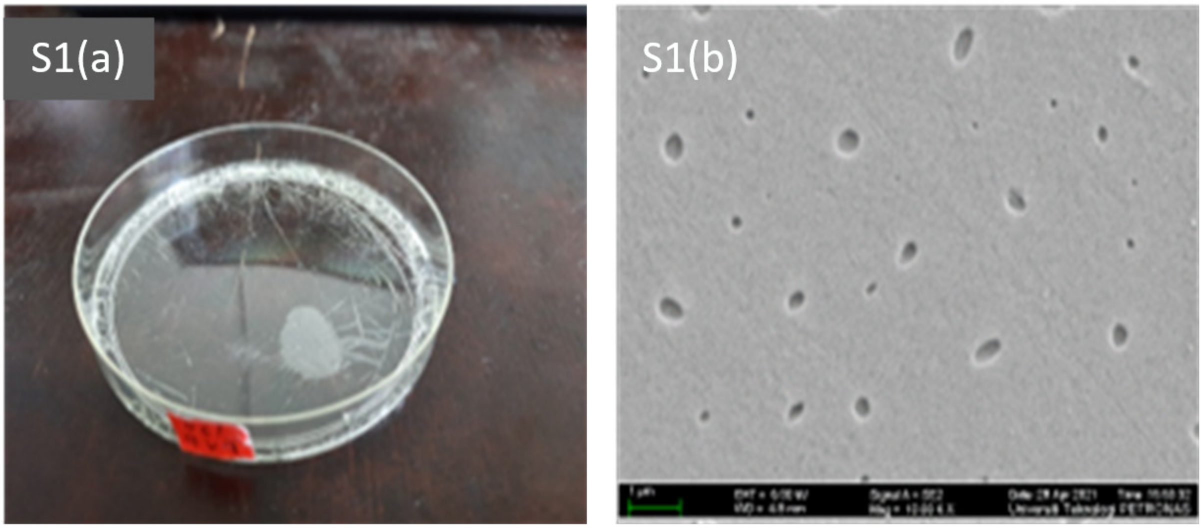

Figure S1. (**a**) Pure PVP film stuck to the petri dish (**b**) FESEM image of pure PVP film on petri dish substrate

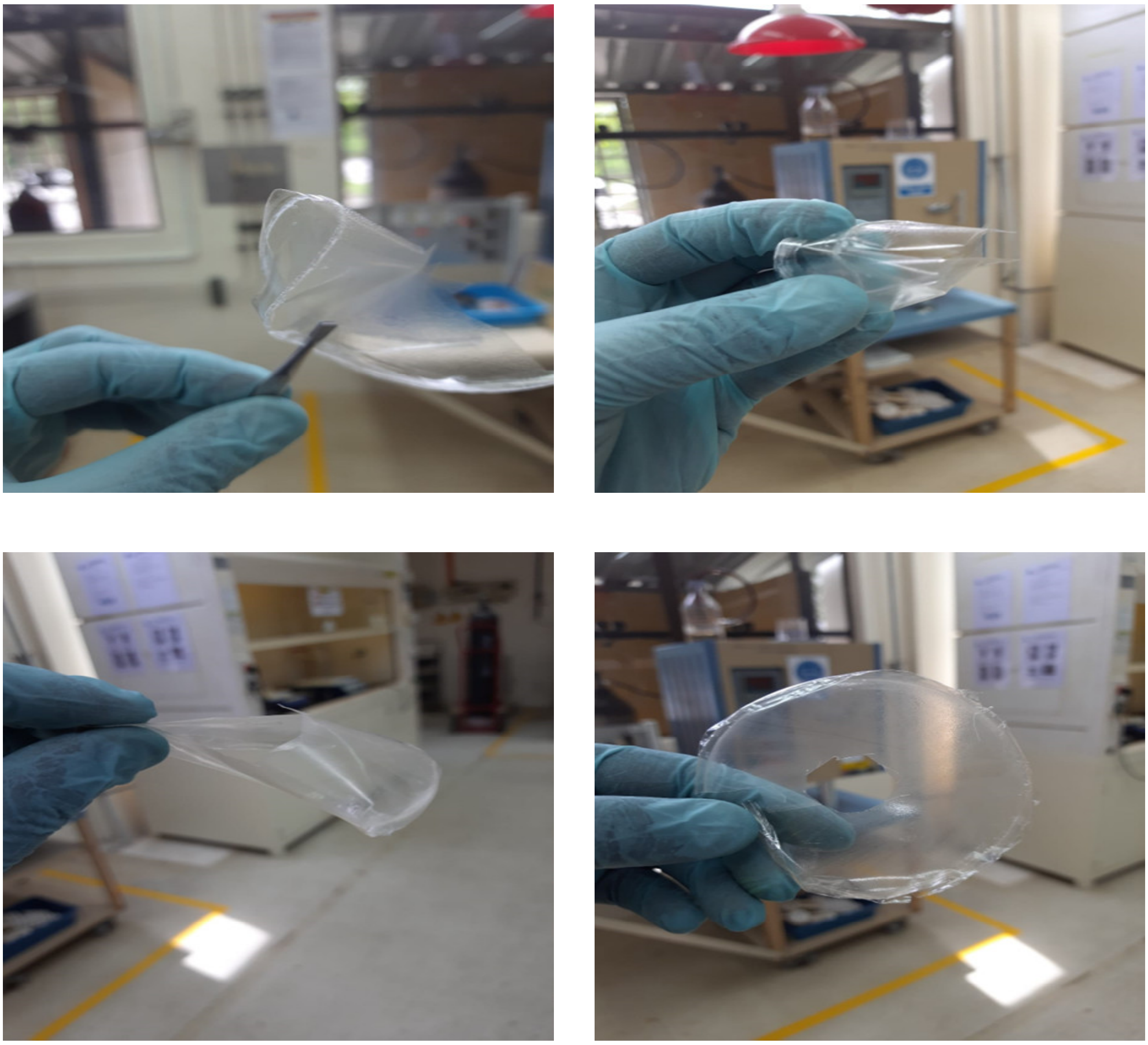

Figure S2. Images of SPEs under different manipulations

Table S1. Band assignment for MC/PVP polymer blend

**Samples**	**Band assignments for MC/PVP films**					**Ref**
	O-H stretching	C-H in CH_2 stretching	C=O stretching	C-N stretching	C-O-C (ether) stretching	
MP100	-	2918	1614	1444 and 1495	-	[1-3]
MP00	3479	2907	-	-	1057	[4-6]
MP10	3436	2923	1664	1444 and 1491	1053	[7, 8]
MP20	3456	2919	1660	1440 and 1286	1049	[9, 10]
MP30	3460	2911	1657	1425 and 1282	1056	[3, 11]
MP40	3463	2899	1654	1422 and 1281	1056	[6, 12]
MP50	3484	2887	1650	1422 and 1278	1064	[2, 9]

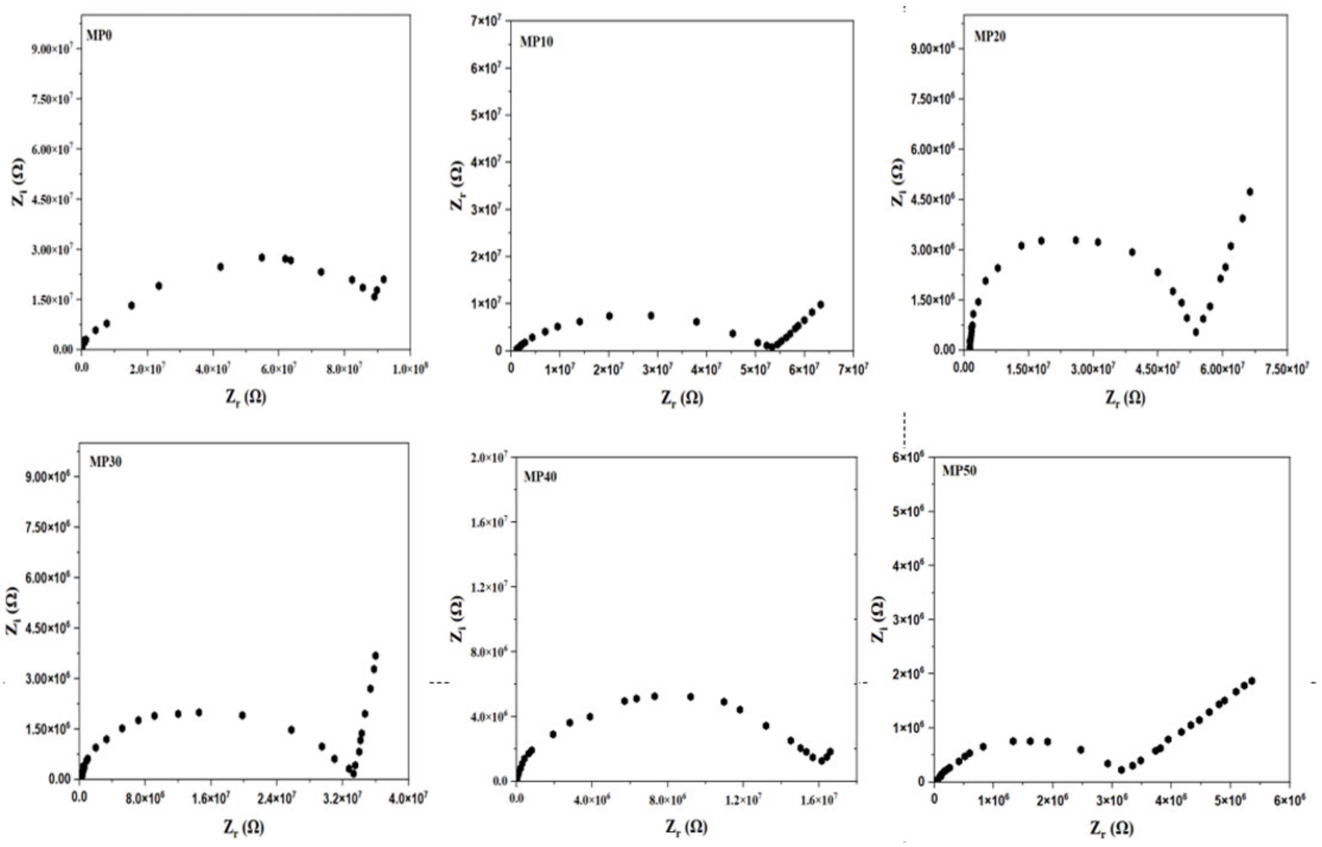

Figure S3. Nyquist plots of MC/PVP polymer blend samples

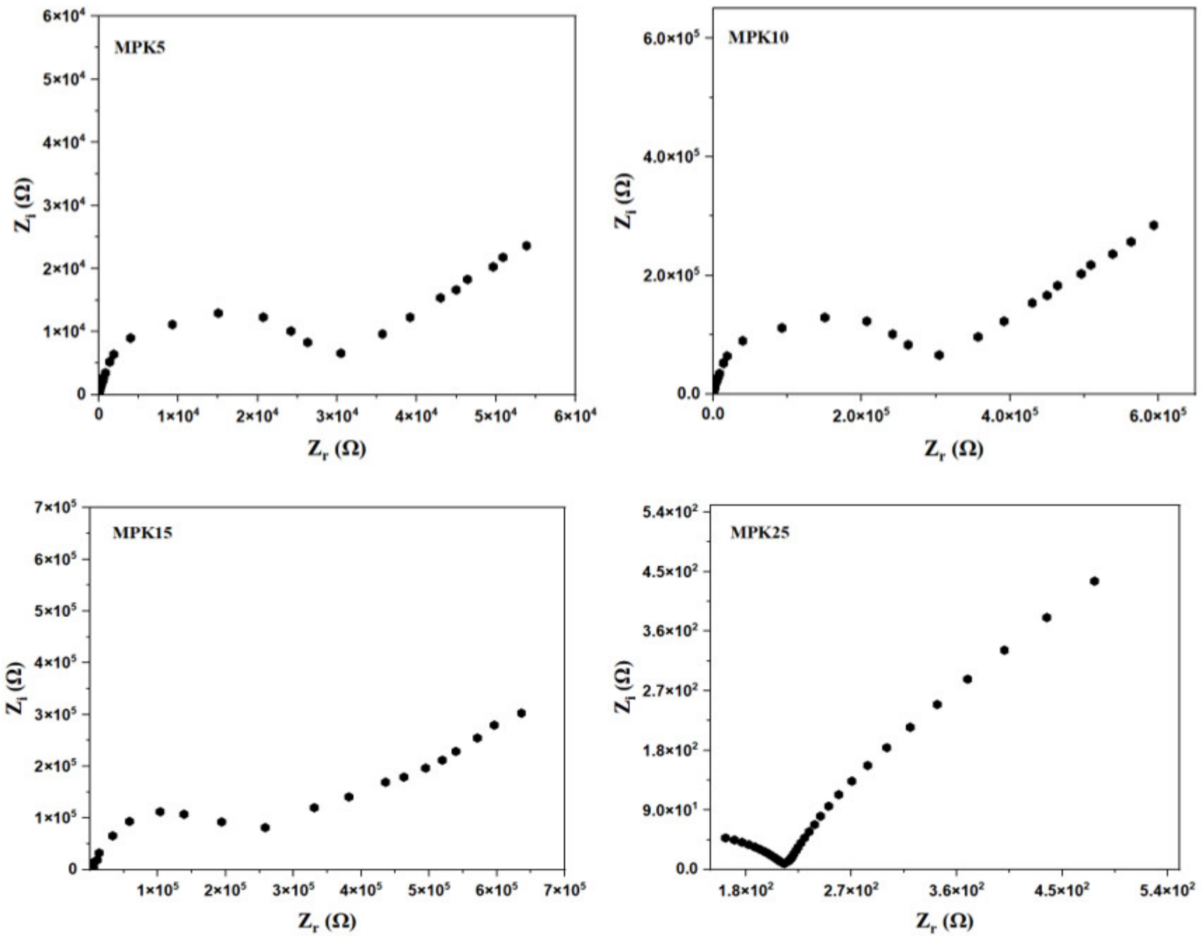

Figure S4. Nyquist plots of other MC/PVP/K_2CO_3 SPEs

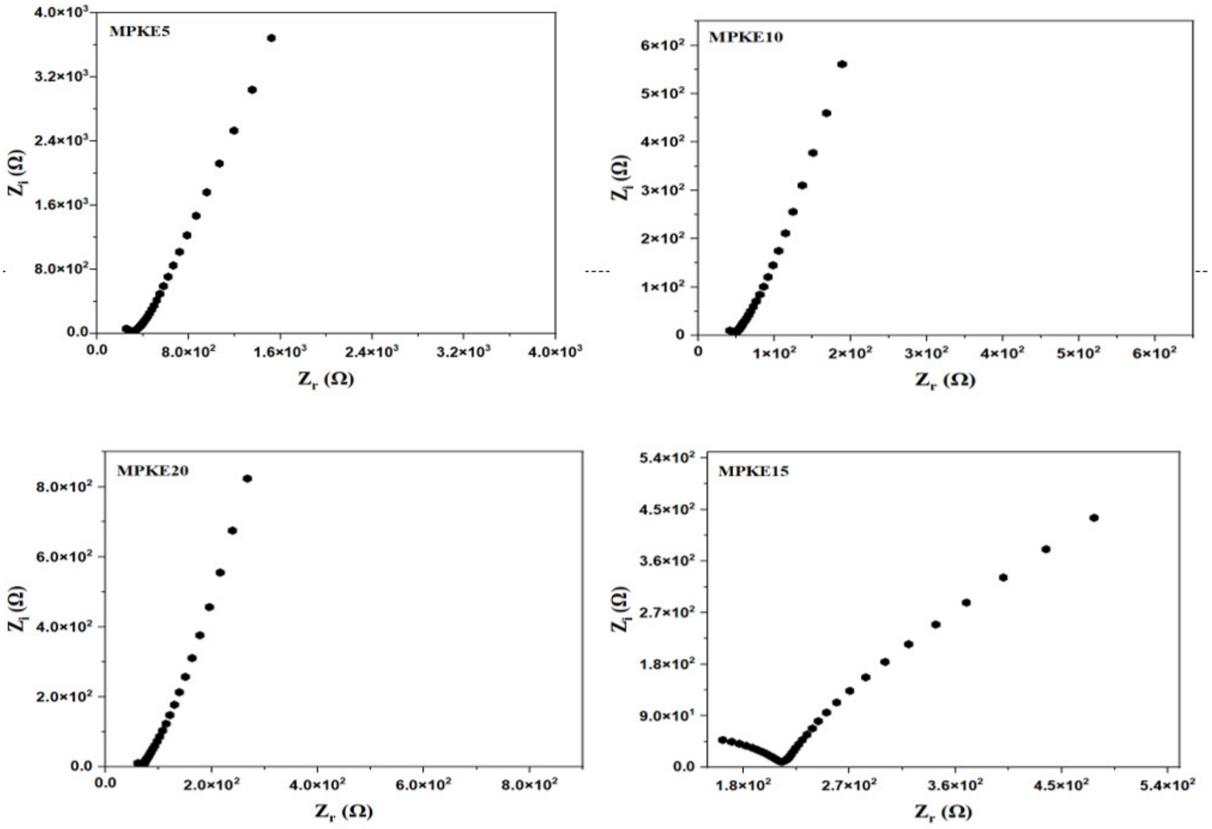

Figure S5: Nyquist plots of other MC/PVP/K_2CO_3/EC SPEs

References

1. Anilkumar, K., et al., *Poly (ethylene oxide)(PEO)–Poly (vinyl pyrrolidone)(PVP) blend polymer based solid electrolyte membranes for developing solid state magnesium ion cells.* 2017. **89**: p. 249-262.
2. Jothi, M.A., et al., *Investigations of lithium ion conducting polymer blend electrolytes using biodegradable cornstarch and PVP.* 2020. **580**: p. 411940.
3. Regu, T., et al., *Proton transport and dielectric properties of high molecular weight polyvinylpyrrolidone (PVP K90) based solid polymer electrolytes for portable electrochemical devices.* 2019. **30**(12): p. 11735-11747.
4. Kadir, M.F.Z., et al., *Biopolymeric electrolyte based on glycerolized methyl cellulose with NH4Br as proton source and potential application in EDLC.* Ionics, 2018. **24**(6): p. 1651-1662 DOI: 10.1007/s11581-017-2330-4.
5. Shamsuri, N.A., et al., *Effect of ammonium thiocyanate on ionic conductivity and thermal properties of polyvinyl alcohol–methylcellulose–based polymer electrolytes.* Ionics, 2020. **26**(12): p. 6083-6093 DOI: 10.1007/s11581-020-03753-9.
6. Nadirah, B.N., et al., *Structural and conductivity studies of polyacrylonitrile/methylcellulose blend based electrolytes embedded with lithium iodide.* International Journal of Hydrogen Energy, 2020. **45**(38): p. 19590-19600 DOI: https://doi.org/10.1016/j.ijhydene.2020.05.016.
7. Ambika, C., et al., *Effect of dimethyl carbonate (DMC) on the electrochemical and cycling properties of solid polymer electrolytes (PVP-MSA) and its application for proton batteries.* Solid State Ionics, 2018. **321**: p. 106-114 DOI: https://doi.org/10.1016/j.ssi.2018.04.013.

8. Aziz, S.B., et al., *Compatible solid polymer electrolyte based on methyl cellulose for energy storage application: Structural, electrical, and electrochemical properties.* 2020. **12**(10): p. 2257.
9. Vahini, M. and M. Muthuvinayagam, *Synthesis and electrochemical studies on sodium ion conducting PVP based solid polymer electrolytes.* Journal of Materials Science: Materials in Electronics, 2019. **30**(6): p. 5609-5619 DOI: 10.1007/s10854-019-00854-8.
10. Aziz, S.B., et al., *Protonic EDLC cell based on chitosan (CS): Methylcellulose (MC) solid polymer blend electrolytes.* 2020: p. 1-12.
11. Abdullah, S., et al. *Characterization of Solid Polymer Electrolyte Membrane made of Methylcellulose and Ammonium Nitrate*. in *Journal of Physics: Conference Series*. 2020. IOP Publishing.
12. Kumar, M.S. and M.C. Rao, *Effect of Al2O3 on structural and dielectric properties of PVP-CH3COONa based solid polymer electrolyte films for energy storage devices.* Heliyon, 2019. **5**(10): p. e02727 DOI: https://doi.org/10.1016/j.heliyon.2019.e02727.
